# Association of anti‐gangliosides antibodies and anti‐CMV antibodies in Guillain–Barré syndrome

**DOI:** 10.1002/brb3.690

**Published:** 2017-04-07

**Authors:** Lijuan Wang, Chunqing Shao, Chunjiao Yang, Xixiong Kang, Guojun Zhang

**Affiliations:** ^1^ Department of Clinical LaboratoryBeijing Tiantan HospitalCapital Medical UniversityBeijingChina; ^2^ China National Clinical Research Center for Neurological DiseasesBeijingChina; ^3^ Monogenic Disease Research Center for Neurological DisorderBeijingChina

**Keywords:** anti‐gangliosides antibody, Cytomegalovirus, Guillain–Barré syndrome

## Abstract

**Introduction:**

Numerous types of infection were closely related to GBS, mainly including *Campylobacter jejuni*, Cytomegalovirus, which may lead to the production of anti‐gangliosides antibodies (AGA)**.** Currently, although there are increased studies on the AGA and a few studies of anti‐CMV antibodies in GBS, the association between them remains poorly documented. Therefore, our research aims to analyze the correlation of anti‐CMV antibodies and AGA in GBS.

**Methods:**

A total of 29 patients with GBS were enrolled in this study. The CMV antibodies were tested by the electrochemiluminescence immunoassay “ECLIA” (Roche Diagnostics GmbH). The serum gangliosides were determined by The EUROLINE test kit.

**Results:**

Of the 29 patients with GBS, 9 (31%) were AGA‐seropositive, in which 22 were CMV‐IgG positive in CSF at the same time, but all 29 samples were CMV‐IgM negative in both serum and CSF. In the AGA‐positive group, the rate of both serum and CSF positive was 87.5% (7/8), higher than 50% (7/14) of the negative group, although no statistical significance was found. In addition, we found that there was a trend of higher ratio of men, a younger age onset, less frequent preceding infection, a higher level of CSF proteins, and less frequent cranial nerve deficits, although the data did not reach a statistical significance.

**Conclusion:**

In spite of no statistical significance association was found between serum AGA and CMV‐IgG in serum and CSF. However, we found that there was a trend of high positive rate of both serum and CSF‐CMV‐IgG in AGA‐positive than the negative group. So we should further expand the sample size to analyze the association between AGA and CMV or other neurotropic virus antibodies in various diseases, to observe whether they could be serological marker of these diseases (especially GBS) or the underlying pathogenesis.

## 1 Introduction

GBS is an immune‐mediated disorder in the peripheral nervous system, characterized by a group of heterogeneous and different clinical, electrophysiological, and pathological findings (Du et al., 2015; Jacobs, Meulstee, van Doorn, & van der Meche, 1997; van der Meche, Meulstee, Vermeulen, & Kievit, 1988). The annual incidence rate of GBS is estimated at 1.1 to 1.8 in each 100,000 persons (Rajabally & Uncini, 2012). It represents series of different subtypes, including acute inflammatory demyelinating polyneuropathy (AIDP), acute motor axonal neuropathy (AMAN), acute motor‐sensory axonal neuropathy (AMSAN), Miller‐Fisher syndrome (MFS) and other relatively rare sorts (Zhang, Wu, Wu, & Zhu, 2014).

Gangliosides are a family of sialylated glycosphingolipids located in higher density in nervous system, especially in axons of neuron (Ledeen & Yu, 1982; Schuster & Haller, 1990). It consists of several subtypes depending on the number and position of sialic acids, the number of glucose molecules, and their synthetic pathways, for example, GM1, GM2, GM3, GD1a, GD1b, GT1b, and GQ1b and so on (Asthana et al., 2016; Yuki, 2012). It is reported that the GBS is associated with various types of infection (such as *Campylobacter jejuni*, Cytomegalovirus, Epstein‐Barr virus, *Mycoplasma pneumoniae*, and hepatitis E virus) which lead to a cross‐reaction with nervous system, demyelination of neurons, and finally initiation of nervous signs and symptoms by stimulating immune system (van Doorn & Jacobs, 2016; Taheraghdam et al., 2014). Simultaneously, accumulating evidence has indicated that the antecedent infection with *C. jejuni* enteritis may trigger the generation of AGA (Nyati & Nyati, 2013). Moreover, previous studies have shown that Cytomegalovirus (CMV), a member of the β herpes family may lead to the incidence of GBS and is second only to *C. jejuni* enteritis (Orlikowski et al., 2011; Taheraghdam et al., 2014). Currently, although there are a number of studies on the AGA and a few studies of anti‐CMV antibodies in GBS, the association between them remains poorly documented (Annunziata, Figura, Galli, Mugnaini, & Lenzi, 2003; McCombe, Wilson, & Prentice, 1992; Taheraghdam et al., 2014). Therefore, our own research aims to analyze the correlation of anti‐CMV antibodies and AGA in the GBS.

## 2 Materials and Methods

### 2.1 Patients

A total of 29 patients with GBS were enrolled in this study from the Laboratory diagnosis center of Beijing Tiantan Hospital, Capital Medical University between October 2012 and December 2013. All patients met the diagnostic criteria of GBS and patients with a fever or infection were excluded (van Koningsveld et al., 2007). All 29 serum samples were selected for the measurement of CMV‐IgG and IgM, IgG AGA, of which 22 CSF specimens were tested for CMV‐IgG. Moreover, a total of 441 other nervous diseases (Peripheral neuropathy 28, Multiple sclerosis 18, Myelopathy 31, Demyelinating disease 67, Viral meningitis 12, Viral encephalitis 19, Epilepsy 58, Cavernous sinus syndrome 9, Acute disseminated encephalomyelitis 8, Intracranial infection 47, Intracranial venous sinus thrombosis 30, Intracranial space‐occupying lesions 37, Cerebral infarction 23, Optic nerve myelitis 15, Motor neuron disease 3, Symptomatic epilepsy 36) from Beijing Tiantan Hospital between January 2015 and December 2015 were analyzed.

### 2.2 Detection of anti‐gangliosides antibodies

**Figure 1:**
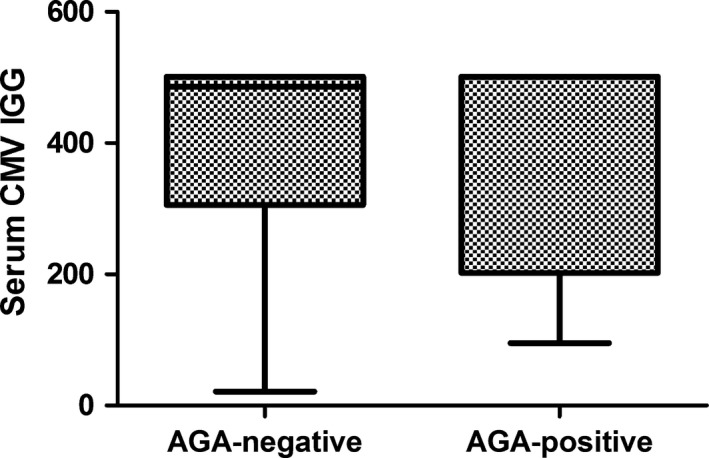
GBS was grouped by AGA positive and negative. Median serum CMV‐IgG levels were 389.41 and 386.1 U/ml for the groups of AGA positive group and negative group, respectively, and there were no significant differences between them (*p *> .05)

We detected auto‐antibodies of the IgG and IgM class to the seven gangliosides GM1, GM2, GM3, GD1a, GD1b, GT1b, and GQ1b in serum by The EUROLINE test kit. By using a combination of different antigens on one strip, multiple auto‐antibodies against gangliosides can be investigated in one sample simultaneously. The test kit contains test strips coated with parallel lines of purified antigens (Figure 1). The patient samples for analysis are diluted 1:51 with ready for use diluted sample buffer. Because of the special membrane used in the present EUROLINE, a pretreatment of the test strips is not necessary. Detailed steps are as follows: (1) Fill each channel with 1.5 ml of the diluted samples and incubate for 120 min at room temperature (+18°C to +25°C) on a rocking shaker with the test strips fully covered with liquid and not float on top; (2) Aspirate off the liquid from each channel and wash 3 × 5 min each with 1.5 ml working strength wash buffer on a rocking shaker; (3) Pipette 1.5 ml diluted enzyme conjugate (alkaline phosphatase conjugated anti‐human IgG/IgM) into each channel and incubate for 60 min at room temperature (+18°C to +25°C) on a rocking shaker; (4) Aspirate off the liquid from each channel and wash as described above; (5) Pipette 1.5 ml substrate solution into the channels of the incubation tray and incubate for 10 min at room temperature (+18°C to +25°C) on a rocking shaker; (6) Aspirate off the liquid from each channel and wash each strip 3 × 1 min with deionized or distilled water; (7) Place test strip on the evaluation protocol, air dry, and evaluate.

### 2.3 Detection of anti‐CMV antibodies

The electrochemiluminescence immunoassay “ECLIA” is used for the measurement of CMV‐IgG/IgM. The first step: 20 μl of sample, biotinylated recombinant CMV‐specific antigens, and CMV‐specific recombinant antigens labeled with a ruthenium complexes) form a sandwich complex. The second step: After addition of streptavidin‐coated microparticles, the complex becomes bound to the solid phase via the interaction of biotin and streptavidin. The reaction mixture is aspirated into the measuring cell where the microparticles are magnetically captured onto the surface of the electrode. Unbound substances are then removed with ProCell/ProCell M. Application of a voltage to the electrode then induces chemiluminescent emission which is measured by a photomultiplier. Results are determined via a calibration curve which is instrument‐specifically generated by 2‐point calibration and a master curve provided via the reagent barcode.

### 2.4 Statistical analysis

Statistical analysis was performed using SPSS 20.0. With respect to the clinical features of the patients with GBS, differences in the proportions between groups were tested using the chi‐square test or Fisher's exact test, and differences in medians were tested using the *t*‐test or nonparametric test. The level of statistical significance was set at *p *< .05.

## 3 Results

**Figure 2:**
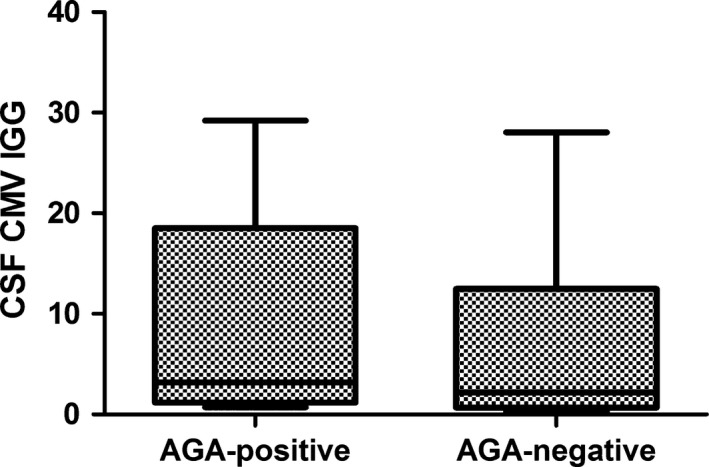
GBS was grouped by AGA positive and negative. Median CSF CMV‐IgG levels were 8.61 and 6.71 U/ml for the groups of AGA positive group and negative group, respectively, and there were no significant differences between them (*p *> .05)

**Figure 3:**
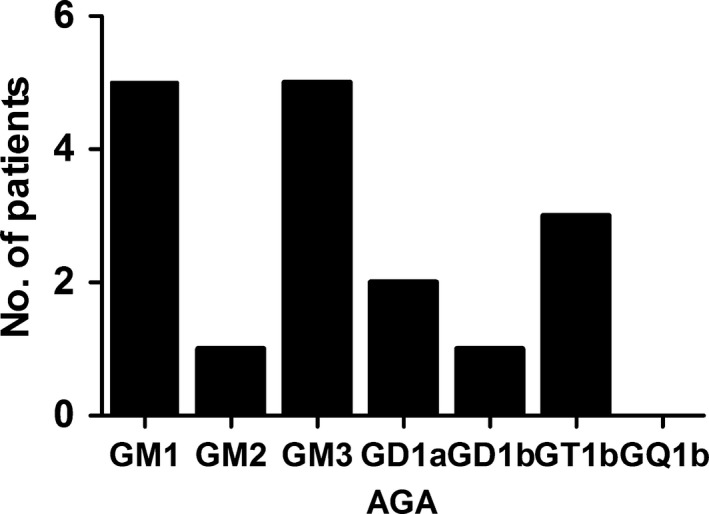
Various kinds of AGA were detected in patients with GBS. The most frequent type was anti‐GM3 and GM1, followed by anti‐GT1b. Anti‐GQ1b antibody is detected in no patients with GBS. (immunoglobulin M‐type and G‐type AGA were considered together in this figure)

Of 29 patients with GBS, 20 (69%) were from men. The age range was 16–75 (median 48.6 years). All were tested for the IgG and IgM of AGA in both serum and CSF. Moreover, the IgG and IgM of CMV in the serum were detected, and 22 of which were selected for the measurement of CSF CMV‐IgG. 9 (31%) of the 29 patients with GBS were AGA‐seropositive. All 29 serum samples were detected CMV‐IgG positive and of which 14 were positive in CSF, companying CMV‐IgM negative in both serum and CSF. Among 22 samples which were measured for the CMV‐IgG in both serum and CSF, 7 (87.5%) were both positive, 1 (12.5%) were only serum positive in AGA‐positive group. The proportion between the both positive and the only serum positive are half and half in the AGA‐negative group (Table 1). However, there were no significant statistically in the AGA‐positive and –negative group (*p *= .167). Meanwhile, there was insignificant difference between serum/CSF CMV‐IgG and AGA in GBS, with *p *= .792/.374 respectively (Figures 1 and 2), and there was no correlation between them (*p *= .079). Various kinds of AGA (including anti‐GM1, anti‐GM2, anti‐GM3, anti‐GD1a, anti‐GD1b, anti‐GQ1b, and anti‐GT1b) were detected in patients with GBS. The percent of IgG antibodies to GM1, GM2, GM3, GD1a, GD1b, GT1b, and GQ1b were 13.8%, 0.0%, 3.4%, 3.4%, 3.4%, 3.4%, and 0.0%. And the type of IgM respectively were 3.4%, 3.4%, 13.8%, 3.4%, 0.0%, 6.9%, 0.0% (Table 2). When immunoglobulin M‐type and G‐type AGS were considered together, the most frequent type was anti‐GM3 and GM1, followed by anti‐GT1b. Anti‐GQ1b antibody is detected in no patients with GBS. In contrast, the most common was the type of IgG to GM1 and IgM to GM3, followed by anti‐GT1b IgM (Figure 3). Clinical features of 9 AGA‐seropositive patients were given in Table 3. From Table 4, no significant differences in gender, age, cranial nerve deficits, and CSF protein between AGA‐positive and –negative group (all *p *> .05). Furthermore, we found that 229 (51.9%) other nervous diseases were CMV‐IgG positive in CSF, which was higher than GBS (48.3%).

**Table 1:** Comparison of CMV‐ IgG between patients with or without AGA

Anti‐CMV‐IgG	AGA –positive (%)	AGA –negative (%)	Total
S (+) C (+)	7 (87.5)	7 (50)	14
S (+) C (−)	1 (12.5)	7 (50)	8
Total	8	14	22

**Table 2:** Comparison of the type of IgG and IgM in different AGA

	IgG class	IgM class
Types of antibodies	No. of patients (%)	No. of patients (%)
Anti‐GM1	4 (13.8)	1 (3.4)
Anti‐GM2	0 (0.0)	1 (3.4)
Anti‐GM3	1 (3.4)	4 (13.8)
Anti‐GD1a	1 (3.4)	1 (3.4)
Anti‐GD1b	1 (3.4)	0 (0.0)
Anti‐GT1b	1 (3.4)	2 (6.9)
Anti‐GQ1b	0 (0.0)	0 (0.0)

**Table 3:** Clinical characteristics of the 9 patients with GBS who were seropositive for AGA

Patients No.	Age(year) /sex	Preceding infection	Muscle strength	Cranial nerve abnormalities	Classification/severity	CSF protein (mg/dL)
1	30/male	Y	Limb 5	/	AIDP/serious	47
2	58/male	N	Upper limb 2Lower limb 1	/	AMAN/serious	106
3	36/male	N	Upper limb 1 Lower limb 2	VII	AMAN/serious	41.5
4	47/male	Y	Limb 4	/	AMAN/mild	98.2
5	62/female	Y	Upper limb 0 Lower limb 1	VII	GBS/ serious	78.6
6	60/male	N	Limb 5	/	CIDP/mild	54.76
7	27/male	N	Limb 4	/	AMAN/ mild	59.1
8	49/female	N	Limb 4	VII	CIDP/mild	208.1
9	45/male	Y	Limb 5	III, IV, VI, VII, IX, X	AIDP/mild	123.2

“/” denotes not carnial nerve abnormalities; “Y” denotes preceding infection and “N” represents not preceding infection; When distal and proximal muscle strength is different, computing the lower muscle strength results.

**Table 4:** Comparison of the clinical characteristics of patients with GBS between the AGA–positive and –negative group

	Positive (*n *= 9)	Negative (*n *= 20)	*p*
Proportion of men	7/9 (77.8%)	13/20 (65%)	.675
Age (years)	46 ± 4.288	49.8 ± 3.801	.556
Preceding infection	4/9 (44%)	10/20 (50%)	1.0
Cranial nerve deficits	4/9 (44%)	13/20 (65%)	.422
CSF protein (mg/dl)	90.7 ± 14.4	70.7 ± 9.17	.272

Except where specified otherwise, the data are *n* (%) or mean ± *SD* values.

## 4 Discussion

GBS, known as a common cause of acute flaccid paralysis, typically occurs after an antecedent infection. Thereafter, it will produce the AGA against the bacterial lipo‐oligosaccharide which cross‐react with gangliosides at nerve membranes, finally leading to demyelinization and axonal degeneration (van den Berg et al., 2014; van Doorn & Jacobs, 2016). Among various microbial infections, *Campylobacter jejuni* is the most common reason to cause GBS and a second infection associated with the GBS is CMV. Elevated researches showed that *Campylobacter jejuni* was closely related to the GBS (Koga, Yuki, & Hirata, 1999; Odaka, Koga, Yuki, Susuki, & Hirata, 2003; Ogawara et al., 2000; Zhang et al., 2010, 2015). Furthermore, serials of investigations indicated that AGA can be found in the serum of patients with CMV‐IgG positive (Caudie et al., 2002; Sivadon et al., 2005; Yuki, Yoshino, Sato, & Miyatake, 1990). Meanwhile, Simanek AM and co‐workers provided evidence that the CMV reaction had the relationship with chronic inflammation (Simanek et al., 2011). Therefore, we investigated the relationship between AGA and CMV‐IgG in patients with GBS in our study.

Our results showed that all 29 patients with GBS was CMV‐IgG seropositive and among them 14 was positive in CSF, yet both serum and CSF CMV‐IgM were negative, which indicated that the 29 patients with GBS were not in acute cytomegalovirus infection phase. And among them, only a small percentage of patients (nine patients) were AGA‐positive, including GM1, GM2, GM3, GD1a, GD1b, and GT1b antibody, with anti‐GQ1b negative in IgM and IgG class. However, only 5–22% CMV infections was found in patients with GBS by Esteghamati, Gouya, Keshtkar, & Mahoney (2008), which is much lower than the results of our study. One possible explanation for this phenomenon is that not all patients with CMV‐IgG‐positive were really CMV infection and the CMV‐DNA in the blood should be detected further. The other reason may be the result of different detection methods. Moreover, the positive rate of CSF CMV‐IgG (48.3%) in patients with GBS is less than that in other nervous diseases (51.9%), indicating that CSF CMV‐IgG could not distinguish between GBS and other neurological diseases. Serum AGA can be found in 14% and 13.3% of the patients with GBS in the study conducted by Hao Q, Aliakbar T and their colleagues respectively (Hao et al., 1998; Taheraghdam et al., 2014). But 31% (9/29) of AGA was found in patients involved in our study, which is higher than the results of them, but lower than the values reported by other investigators (van den Berg et al., 2014; van Doorn & Jacobs, 2016). In addition, our results are not in accordance with those published by Jacobs, van Doorn, Groeneveld, Tio‐Gillen, & van der Meche (1997), Jacobs et al. (1998). They reported that anti‐GM2 IgM antibodies were found more often in patients with GBS with CMV infection (22%) than in patients without the infection. However, in our study, 22 serum samples obtained from patients with GBS has a positive result of CMV‐IgG of which 14 also reveals CSF‐positive, with only 1 of the anti‐GM2‐IgM positive. Moreover, Irie et al. (1996) found that a lower rate of CMV infections in their patients, in which they obtained the serum samples at rather a long time after neurological onset. Additionally, Jacobs et al. (1997) also showed that anti‐GM2 antibodies can be found in some patients with GBS with *C. jejuni* infections, yet, the frequency was significantly lower than in patients infected with CMV. Interestingly, some AGA specificities are associated with the GBS subtypes such as anti‐GM1 is closely related to the AMAN and GQ1b antibody are notably associated with MFS, characterized by ophthalmoplegia, ataxia, and areflexia (van Doorn & Jacobs, 2016; Mori, Kuwabara, & Yuki, 2012). Researchers have shown that approximately up to 80% GQ1b antibody was found in patients with MFS (Ito et al., 2008; Mori et al., 2012). However, in our study, no GQ1b antibody was measured, although there are the patients diagnosed as MFS. Possible reason may occur as a result of the detection method used by us. Hashemilar et al. (2014) suggested that EUROLINE method could be used instead of the ELISA method except for the anti‐GQ1b antibody.

In this study, we confirmed that there was a trend of higher ratio of men, a younger age onset, less frequent preceding infection, a higher level of CSF proteins, less frequent cranial nerve deficits, although the data did not reach a statistical significance. Moreover, a higher positive rate of CMV‐IgG both in the serum and CSF was found in AGA‐positive group than the negative group, but no statistical significance was found. This result may probably because the small samples are included in the previous studies. However, Fan et al. (2017) suggested that facial nerve palsy was connected to the presence of IgM‐AGA. Only 48.3% (14/29) of patients have symptoms of a respiratory or gastrointestinal tract infection before the onset of GBS which was found in our articles, <two‐thirds described by van den Berg et al. (2014).

The present study still has several limitations. On the one hand, a small research specimen size was included in our study. On the other hand, we just analyzed the association between anti‐CMV antibodies and AGA in patients with GBS. Actually, we found that there may be a connection between neurotropic virus antibodies (for instance CMV, Epstein‐Barr virus, *M. pneumoniae*, Herpes simplex virus) and other nervous system diseases, such as multiple sclerosis, demyelinating disease, peripheral neuropathy, and neuro‐opticmyelitis and so on by retrospective analysis. Moreover, our results showed there was a trend of high positive rate of both serum and CSF CMV‐IgG in AGA‐positive than the negative group in GBS. So we should further expand the sample size to analyze the association between AGA and CMV, EB or other neurotropic virus antibodies in various nervous system diseases, to observe whether they could be serological marker of these diseases or the underlying pathogenesis.

## Conflict of Interest

None declared.
